# The culture of winemaking as a protective factor against at-risk drinking: a peer-led intervention with adolescents

**DOI:** 10.1192/j.eurpsy.2023.718

**Published:** 2023-07-19

**Authors:** E. Rossero, A. Barbieri

**Affiliations:** ^1^Eclectica+ Research and Training, Turin; ^2^Mental Health Department, ASL CN1, Cuneo, Italy

## Abstract

**Introduction:**

Alcohol consumption is part of the global youth culture and represents a dimension of young people’s social identity. Even if at-risk drinking in the young population is lower in Italy compared to other countries, the increasing complexity and changes in global values may influence risky behaviours, which therefore require attention and preventive strategies.

**Objectives:**

The intervention followed a pilot named *The Vineyard Project*, which engaged a group of young mental health service users in local practices of hand-harvesting grape. The initiative was hosted in the wine-producing area of Langhe (Italy), which shows lower rates of at-risk drinking also due to the protective role played by the cultural dimension of winemaking craft. The pilot inspired a peer led-intervention in a local high school, acknowledging the crucial role that educational settings play in the lives of young people and the relevance of peer influence on adolescents’ behaviours.

**Methods:**

Semi-structured interviews with young people participating in *the Vineyard Project* have been conducted, audio-recorded and shared with high school students (n=80) to become the object of dedicated workshops. Interviews explored participants’ experience in the vineyard, the relationship they developed with the vines and with professional vine growers, their role in the winemaking process, and the emotional and sensorial contents of their immersion in the viticultural landscape.

**Results:**

The peer-led intervention showed promising results in producing benefits beyond the group of young people directly involved in the vineyard activities. By narrating their experiences through the interviews, participants acted as cultural mediators with the students who subsequently listened to their stories. Their narratives represent unusual accounts of the world of wine and its production, drawing on the perspective of non-expert, young people that are not familiar with the viticultural landscape. The embodied knowledge they could learn from professional vine growers, concerning the harvest as well as other activities of care for the vines, contribute to portray wine as a cultural product, which is the result of traditions handed down from generation to generation, hard work, competent interventions on the vines and the other living beings of the vineyard. This unusual perspective on wine was perceived as particularly surprising for students who lived in the area, who became so acquainted with the viticultural landscape and the discourses around its products to the point of taking it for granted in a non-reflexive attitude.

**Conclusions:**

Findings suggest that peer-led interventions concerning wine and other drinking products, narrated in their cultural dimension (e.g. their story, identity, local traditions, practices and the tacit knowledge implied in their production), may encourage a limited and competent consumption among young people.

**Disclosure of Interest:**

None Declared

